# Casting Without Reduction Versus Closed Reduction With or Without Fixation in the Treatment of Distal Radius Fractures in Children: Protocol for a Randomized Noninferiority Trial

**DOI:** 10.2196/34576

**Published:** 2022-04-14

**Authors:** Maria Fernanda Garcia-Rueda, Adriana Patricia Bohorquez-Penaranda, Jacky Fabian Armando Gil-Laverde, Francisco Javier Aguilar-Sierra, Camilo Mendoza-Pulido

**Affiliations:** 1 Department of Orthopedics Instituto Roosevelt Bogotá Colombia; 2 Department of Clinical Epidemiology and Biostatistics Pontificia Universidad Javeriana Bogotá Colombia; 3 Department of Physical Medicine and Rehabilitation School of Medicine Universidad Nacional de Colombia Bogotá Colombia; 4 Department of Rehabilitation Medicine Instituto Roosevelt Bogotá Colombia

**Keywords:** radius fractures, distal radius, pediatric, remodeling, surgical reduction, cast immobilization, outcome measure

## Abstract

**Background:**

Acute treatment for distal radius fractures, the most frequent fractures in the pediatric population, represents a challenge to the orthopedic surgeon. Deciding on surgical restoration of the alignment or cast immobilization without reducing the fracture is a complex concern given the remodeling potential of bones in children. In addition, the lack of evidence-based safe boundaries of shortening and angulation, that will not jeopardize upper-extremity functionality in the future, further complicates this decision.

**Objective:**

The authors aim to measure functional outcomes, assessed using the Patient-Reported Outcomes Measurement Information System (PROMIS) Pediatric Physical Function v2.0 instrument. The authors hypothesize that outcomes will not be worse in children treated with cast immobilization in situ compared with those treated with closed reduction with or without percutaneous fixation. The authors also aim to compare the following as secondary outcomes: ulnar variance and fracture alignment in the sagittal and coronal planes, range of motion, pressure ulcers, pain control, radius osteotomy due to deformity, pseudoarthrosis cure, and remanipulation.

**Methods:**

This is the protocol of a randomized noninferiority trial comparing upper-extremity functionality in children aged 5 to 10 years, after sustaining a distal radius fracture, treated with either cast immobilization in situ or closed reduction with or without fixation in a single orthopedic hospital. Functional follow-up is projected at 6 months, while clinical and radiographic follow-up will occur at 2 weeks, 3 months, and 9 months.

**Results:**

Recruitment commenced in July 2021. As of January 2022, 23 children have been randomized. Authors expect an average of 5 patients to be recruited monthly; therefore, recruitment and analysis should be complete by October 2024.

**Conclusions:**

This experimental design that addresses upper-extremity functionality after cast immobilization in situ in children who have sustained a distal fracture of the radius may yield compelling information that could aid the clinician in deciding on the most suitable orthopedic treatment.

**Trial Registration:**

ClinicalTrials.gov NCT05008029; https://clinicaltrials.gov/ct2/show/NCT05008029

**International Registered Report Identifier (IRRID):**

DERR1-10.2196/34576

## Introduction

Fractures are prevalent in the pediatric population, with rough estimates of at least one in every three individuals experiencing a fracture before adulthood [1]. Upper-limb fractures, specifically distal metaphyseal radius fractures, are the most frequent, with up to 35% of the fractures occurring during childhood and a significantly increasing trend during the past decades [2-5]. Unimodal age distribution and seasonal association linked to physical activity have been hypothesized [6]. Treatment options include in situ cast immobilization or fracture reduction with or without fixation with Kirschner wires (K-wires); however, deciding on either procedure has long been controversial [7]. As stated by Ploegmakers and Verheyen [8], the decision to accept, reduce, or operate on these kinds of fractures relies on the physician’s experience and gut feelings. Hence, tolerated age-dependent angulation limits were systematically reviewed and presented as the sole criterion used to determine treatment. Alas, the authors declared methodology limitations due to the lack of adequately designed trials. Consequently, even between experienced pediatric orthopedic surgeons, treatment agreement has merely been considered as fair [9].

Distal radius fractures have a remarkable remodeling potential. It has been a long time since Friberg [10] described the intrinsic ability of epiphyseal plates to change inclination in relation to the forearm axis. Remodeling rate is related to angulation: higher rates occur with more severe deformities, and they progressively decrease as the alignment approaches to normality [11]. Observational designs have shown that fractures up to 29° of angulation and 19 mm of shortening immobilized in situ regain complete alignment after a year of follow-up [12]. In another series of children whose fractures were immobilized without restoring the length of the radius, neutral ulnar variance at final follow-up for the whole sample was reported [13].

Furthermore, usual radius morphology is present within 3 years when alignment is lost, even when the fracture is immobilized without any attempt to regain alignment [14]. In contrast, the effect of age on remodeling potential is doubtful. Shorter times to complete alignment have been reported in younger children, while it is believed that remodeling potential decreases toward skeletal maturity [12,15]. Remodeling potential has been recognized in children up to 14 years of age [16].

Percutaneous K-wire fixation has been recommended, but indications vary [17,18]. Classically, wiring should be considered when perfect reduction is not achievable or in the presence of instability or an intact ulna [19,20]. Recent research suggests that operative interventions are the treatment of choice when some angulation and shortening criteria are not met. Nonetheless, more research is needed to identify those who would benefit the most from fixation [21]. Some specialized centers reserve distal radius corrective osteotomy solely for fracture malunion in children approaching the end of growth or associated physeal arrest. It is also the treatment of choice for children with congenital dysplastic conditions of the bone [22].

Upper-extremity functionality is the ability to reach, grasp, and manipulate objects to perform daily life activities [23]. Functionality is a major concern in these fractures, as residual angular deformities of the distal forearm decreases the range for pronation and supination [24,25]. Most of the instruments that assess upper-extremity functionality in the pediatric population are specifically designed for children with longitudinal deficiencies, amputations, or neurodevelopmental delay. Nonspecific instruments and questionnaires have been used as functional outcomes in clinical trials comparing interventions for wrist fractures in children [26-28]. Specific instruments for the adult population have also been published; however, several items refer to tasks that a 5-year-old might not properly execute [29,30].

The physical functioning domain of the Patient-Reported Outcomes Measurement Information System (PROMIS) has been available since 2011 for assessing children’s functionality among those without any disability. It is a precise and easy-to-administer outcome measurement instrument suitable for children with orthopedic conditions. Children who are 8 years of age or older are able to effortlessly answer the questionnaire unaided. A parent-proxy version is also available for younger children. Upper-extremity functionality is a subdomain of the physical functioning domain. It comprises 29 Likert-type questions that inquire about the difficulty of performing activities during the past week [31].

We propose a noninferiority clinical trial where children who sustained a distal fracture of the radius will be randomly allocated, in the acute setting, to either in situ cast immobilization or closed reduction and cast immobilization, with or without K-wire fixation. The trial’s primary goal is to establish whether upper-extremity functionality at 6 months follow-up, measured using the PROMIS physical functioning domain, has not become worse for the former group compared with the latter. Secondarily, range of motion (ROM), alignment, complications, and additional needed treatments will be contrasted.

## Methods

### Study Design and Procedures

A randomized noninferiority single-institution design is proposed. The trial will take place at a children’s hospital that focuses on providing treatment for children with orthopedic and neuromuscular conditions. Following two accepted orthopedic treatments for distal radius fractures, namely cast immobilization in situ or closed reduction and immobilization with or without fixation, functional outcomes, as measured using the PROMIS physical functioning domain, will be compared [14,17,31].

### Patient Selection

Children 5 to 10 years of age with a proven acute (ie, within 14 days after injury) metaphyseal fracture of the distal radius (23-M 2-3 or 23r-M 2-3 AO pediatric classification) will be regarded as eligible. Children will be included, granted that the fracture has either shortening from 0 mm to 10 mm or angulation from 10° to 20° in the oblique plane. Children will be excluded when a simultaneous fracture in the same limb (eg, fracture of the ulna) or a pathologic or open fracture is present, or in the scenario of polytrauma (Injury Severity Score ≥16), neuromuscular or metabolic bone diseases, associated neurologic or vascular lesions, or previous infection or fracture of the fractured radius. Children with longitudinal limb deficiencies will also be excluded.

### Randomization

Randomization will be performed centrally by the institution’s medical research department; therefore, allocation will be concealed to the orthopedic surgeons. The big stick design (BSD) method, with a maximum tolerated imbalance of 2, will be the methodology used for randomization. The BSD method has a very low probability of correctly guessing where the following individual will be allocated compared to other designs [32]. The randomizeR package for R statistical software (version 4.1.0; The R Foundation) will be used [33].

### Interventions

Children will be routinely provided with conventional analgesics in the acute setting prior to orthopedic treatment. Afterward, the principal researcher will invite children and parents to enter the trial upon confirming admission criteria. Informed consent, along with informed assent, will be given. Children will be allocated in a 1:1 ratio to either the experimental or control group. In the former group, the fracture will be immobilized without reduction. In the latter group, the fracture will be reduced and immobilized. In the case of instability, K-wires will be used. Instability is considered when, after reduction, alignment is lost: a new displacement greater than 50% or angulation greater than 10°. General anesthesia will be mandatory for the control group. Discharge within 2 hours is the standard practice for both procedures. Casts and K-wires will be removed after 6 weeks.

### Endpoints and Follow-Up

Children will be evaluated at about 2 and 6 weeks and 3, 6, and 9 months after randomization. The primary endpoint will be upper-extremity functional assessment using the PROMIS Pediatric Physical Function v2.0 instrument at 6 months [31]. Parents will serve as proxies of children younger than 8 years of age. Children 8 years of age or older will answer the questionnaire by themselves.

**Figure 1:**
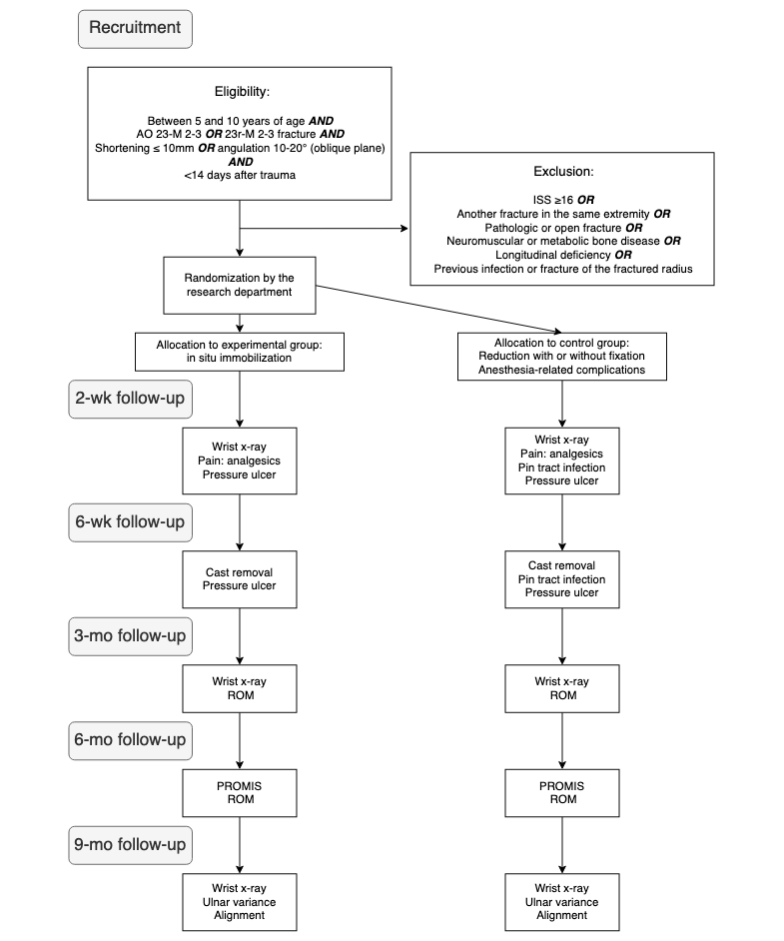
Study design flowchart. ISS: Injury Severity Score; mo: month; PROMIS: Patient-Reported Outcomes Measurement Information System; ROM: range of motion; wk: week.

Secondary outcomes will be ulnar variance and fracture alignment in the sagittal and coronal planes measured in wrist radiographs with a conventional goniometer and ROM. Plain radiographs will be obtained immediately after orthopedic treatment and during follow-up at 2 weeks, 3 months, and 9 months. Additionally, anesthesia-related adverse effects, pressure ulcers according to the National Pressure Ulcer Advisory Panel, the number of days with oral analgesics, and Dahl classification of pin tract infections will also be registered [34]. Additional treatments, such as radius osteotomy due to deformity, pseudoarthrosis cure, and remanipulation, will also be registered. Figure 1 depicts the flow of the study.

### Power Analysis and Sample Calculation

For sample calculations, PROMIS data from children with an upper-extremity fracture were obtained from a recent publication [35]. Assuming a noninferiority threshold of 5 in the physical function domain, a sample of 126 children (63 per group) is required to demonstrate no significant difference between the groups (1-tailed α=.05, β=.2). The sample will be inflated by about 20% to 152, expecting loss to follow-up.

### Data Management

Participants’ information will be entered into REDCap (Research Electronic Data Capture) software. Registries will be password protected, with access granted to the principal researcher and the study coordinator. Data will be kept at the institution’s medical research department. After completing the study, nonanonymized documents will be preserved for 2 years.

### Statistical Analysis

Customary descriptive statistics will be used whether the variables are continuous or categorical. The intention-to-treat principle will be followed. Authors of the scale at Northwestern University will standardize PROMIS scores once data gathering is complete. Afterward, the primary outcome will be evaluated with a *t* test. The noninferiority threshold will be compared to the lower bound of the 95% CI of the mean difference. Continuous outcomes, namely ROM, ulnar variance, fracture alignment, and days with oral analgesics, will be compared with either a *t* test or a Mann-Whitney *U* test conforming to the distribution of the variables. Categorical outcomes, namely anesthesia-related adverse effects, pressure ulcers, pin tract infections, osteotomies, pseudoarthrosis, and remanipulations, will be compared with the Fisher exact test. Early termination of the trial is unlikely, so interim analyses are not being considered; given the proposed design, neither superiority nor futility of the experimental treatment is expected.

### Ethics Approval

The ethics committees from Instituto Roosevelt and Pontificia Universidad Javeriana evaluated and approved the trial’s protocol, research manual, consent and assent forms, and information brochures (approval Nos. 2021012101-002 and FM-CIE-0416-21) in April 2021.

## Results

The authors’ institution will fully fund the trial and all related expenses. No external sources of funding are associated with this trial. The trial was registered at ClinicalTrials.gov (NCT05008029). Recruitment commenced in July 2021. As of January 2022, 23 children have been randomized. At the time of enrollment, the principal researcher personally interviews parents and children, and the trial’s general characteristics are explained fully. Benefits, such as a closer follow-up (eg, radiographs, pain, and functionality), are also presented. Authors expect an average of 5 patients to be recruited monthly; therefore, recruitment and analysis should be complete by October 2024.

## Discussion

This is the protocol for the first randomized trial that compares functional outcomes of nonreduced versus reduced distal radius fractures in children. To the authors’ knowledge, medical literature lacks experimental designs that account for shortening and angulation of this type of fracture in this age group. Cadaveric studies have documented the effects on the motion of the distal radioulnar joint caused by angular deformities related to distal fractures of the radius [25,36]. Necessary pronation and supination ROMs for functional tasks in children and adolescents have also been ascertained with 3D-motion analysis systems [37]. A randomized clinical design would strengthen the evidence of secure shortening and angulation boundaries in terms of functional outcomes and would aid clinicians in the decision-making process in everyday practice.

A strength of this trial is the objective evaluation of the functional outcomes. The PROMIS scale was envisioned with modern measurement theory, which guarantees meaningful statistical results from a Likert-type scale. Further, the PROMIS was explicitly designed for the pediatric population, including previously healthy children who have sustained a wrist fracture [29].

The limitations of this trial are the lack of blinding for evident reasons and generalizability. Children will be recruited and treated in a specialized center. In most institutions in the authors’ country that deal with fractures in children, a pediatric orthopedic surgeon is not accessible. The allocation will be open-labeled for patients, parents, and medical staff. This scenario may affect participants’ feelings of well-being. However, analyses will be blinded. Finally, cost analyses were never an objective for this trial. The expected results may be relevant in terms of costs to health systems.

## Abbreviations

BSD: big stick design
K-wire: Kirschner wire
PROMIS: Patient-Reported Outcomes Measurement Information System
REDCap: Research Electronic Data Capture
ROM: range of motion
